# Assessing the performance of different outcomes for tumor growth studies with animal models

**DOI:** 10.1002/ame2.12250

**Published:** 2022-06-14

**Authors:** Luke W. Patten, Patrick Blatchford, Matthew Strand, Alexander M. Kaizer

**Affiliations:** ^1^ Department of Biostatistics and Informatics University of Colorado Aurora Colorado USA

**Keywords:** mouse model, oncology, outcome selection, patient‐derived xenograft (PDX), statistical analysis, TGII, translational science

## Abstract

The consistency of reporting results for patient‐derived xenograft (PDX) studies is an area of concern. The PDX method commonly starts by implanting a derivative of a human tumor into a mouse, then comparing the tumor growth under different treatment conditions. Currently, a wide array of statistical methods (e.g., *t*‐test, regression, chi‐squared test) are used to analyze these data, which ultimately depend on the outcome chosen (e.g., tumor volume, relative growth, categorical growth). In this simulation study, we provide empirical evidence for the outcome selection process by comparing the performance of both commonly used outcomes and novel variations of common outcomes used in PDX studies. Data were simulated to mimic tumor growth under multiple scenarios, then each outcome of interest was evaluated for 10 000 iterations. Comparisons between different outcomes were made with respect to average bias, variance, type‐1 error, and power. A total of 18 continuous, categorical, and time‐to‐event outcomes were evaluated, with ultimately 2 outcomes outperforming the others: final tumor volume and change in tumor volume from baseline. Notably, the novel variations of the tumor growth inhibition index (TGII)—a commonly used outcome in PDX studies—was found to perform poorly in several scenarios with inflated type‐1 error rates and a relatively large bias. Finally, all outcomes of interest were applied to a real‐world dataset.

## Graphical abstract

The choice of outcome in any animal study is important since it affects the appropriate statistical analysis and the ability to detect a meaningful difference if it exists. In this paper, we introduce various outcomes used in oncology animal models for tumor models and demonstrate the optimal choices based on simulation studies and real world application.

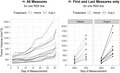

## 1 INTRODUCTION

A common way to assess the efficacy of an anticancer treatment is to analyze the tumor growth in patient‐derived xenografts (PDX).^1^, ^2^, ^3^, ^4^ The PDX approach involves the direct implantation of human tissue samples into mice, where the mice are assigned to treatment groups then tumor volumes are measured over multiple weeks. This direct implantation into mice may allow for more heterogeneity than laboratory‐grown tissue samples,^1^, ^2^, ^5^ and therefore the results may be more directly applicable to human patients.

A PDX study typically assigns many mice to each source of human tissue. For example, one study might have 5 different people contributing tumor samples that are then each allocated to 10 mice (all 10 receiving a distinct sample of that patient's tumor), resulting in a total of 50 mice. For the purpose of this study, we will define one person's set of mice as a PDX line. Continuing with the example, there would be 5 PDX lines with 10 mice each. Studies that use more than 1 PDX line have the potential benefit of adding more real‐world variability (variability across patients) to the study, which may make the results of an antitumor treatment more generalizable.

When quantifying the effect of antitumor treatments, there are a multitude of approaches that a researcher might take. Although these multiple approaches can provide a more individualized approach to each unique study, this may lead to inconsistent reporting of results across studies. One key focus when designing any study should be the selection of the outcome. In a PDX study, the typical question of interest is “is the treatment more effective at reducing tumor growth than a comparator treatment, and if so, by how much?”. There are many potential outcomes that one might choose to answer this, so which outcome is the “optimal” choice? Ideally, the optimal outcome should be chosen primarily on the basis of statistical considerations, especially when there are multiple outcomes that appear to answer the question clinically. As evident by the heterogeneity in the approaches reported in PDX studies,^6^, ^7^, ^8^, ^9^, ^10^, ^11^, ^12^, ^13^ this field lacks a standard approach to outcome selection—justifying the need to investigate an array of potential outcomes. The purpose of this study is to evaluate the performance of different continuous, categorical, and time‐to‐event outcomes with respect to bias, variance, power, and type‐1 error, and to ultimately provide empirical reasoning for outcome selection in PDX studies.

From a statistician's standpoint, the most intuitive outcomes for PDX studies would be the raw tumor volume or the change in tumor volume from baseline. In a cross‐sectional study, this would correspond to the final volume measured and the final difference in volumes (final—initial), respectively. If noncontinuous outcomes were of interest for a given study design, then a binary or categorical variable could easily be computed if biologically meaningful cutoff values for either the final volume or the final difference exist. Another more detailed noncontinuous outcome would be a time‐to‐event outcome, where we could compare summaries such as the median time (in days) until the tumors reach a certain threshold in size, if they do in fact reach that threshold.

Beyond the more intuitive outcomes in the prior paragraph, there exists a range of other possibilities that warrant exploration and rigorous empirical evaluation. First, since the final difference is intuitive, then the final ratio (final volume divided by initial volume) may be of utility as well. Second, the area under the curve (AUC) could be computed using the trapezoidal rule. There are 2 options for the AUC calculation: (1) use only the first and last measurements, or (2) use all timepoints since tumor size is generally evaluated on a set schedule over the course of the study. By using all timepoints, we may be able to capture more information about the tumor growth over the course of the study and potentially differentiate treatments that delay growth even if they have similar volumes at the end of the study. The tumor growth inhibition index (TGII) is commonly used as a summary in PDX studies for each PDX line and is usually calculated on the basis of the ratio of mean tumor volume in different treatment groups. However, if TGII were the outcome for a single PDX line, new definitions with respect to individual mice by treatment would need to be defined and evaluated. With this motivation based on use of TGII in PDX studies, novel variations of the TGII were proposed and evaluated for this article. Since TGII is a ratio, it seems statistically intuitive to also investigate this outcome as the relative difference in tumor growth (using subtraction rather than division), though the clinical interpretation may not be as straightforward. The proposed TGII measures are defined in Section 2.1.

The remainder of this manuscript focuses on identifying the optimal outcomes for use within a single PDX study. Section 2 details the specific outcomes considered, details the simulation study, and introduces the real‐world dataset of a PDX study. Results are summarized in Section 3 for both the simulation study and real‐world results. Sections 4 and 5 include a brief discussion and conclusion.

## 2 MATERIALS AND METHODS

### 2.1 Outcomes of interest

**FIGURE 1:**
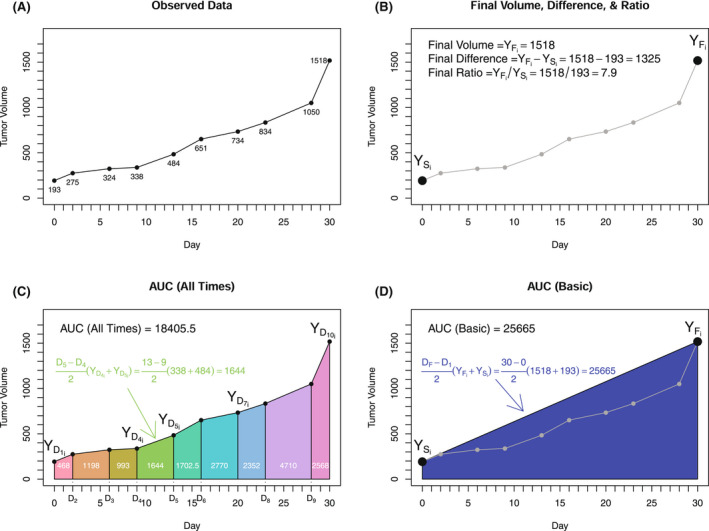
Schematic illustrating how to calculate “individual” outcomes with single tumor data over time (A), calculation of baseline/final measures (B), area under the curve (AUC) for all times (C), and basic AUC using baseline/final measures (D)

**FIGURE 2:**
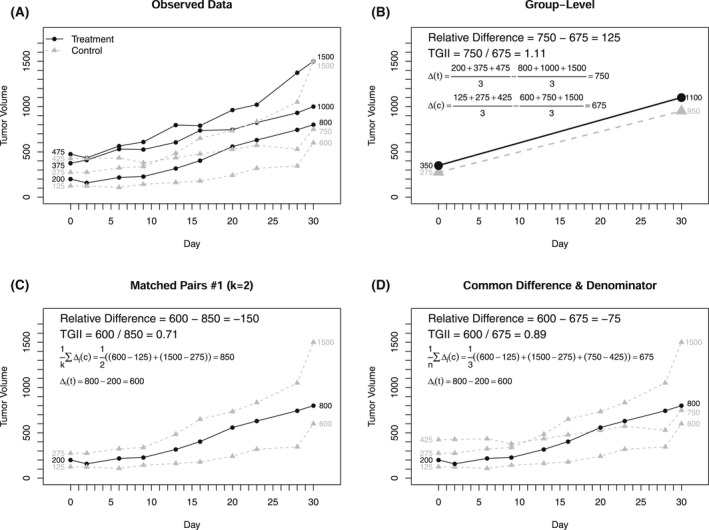
Schematic illustrating how to calculate select “relative” outcomes with three treatment and three control tumors (A), baseline/final changes by group level summary (B), matched pairs for highlighted treatment tumor with k=2 (C), and outcomes based on common differences and denominators (D)

The performance of 18 outcomes (15 continuous, 2 categorical, 1 time to event) was compared via simulations, asymptotic properties, and application to a real‐world PDX study. Conceptually, we classify the 15 continuous outcomes as either “individual” or “relative” outcomes (Table 1). “Individual” continuous outcomes are applied to each tumor, whereas “relative” outcomes are applied to the treated group only because the control group values are already incorporated into the calculations. Figures 1 and 2 present schematics for the calculation of selected outcomes for individual and relative measures, respectively, and are described in greater detail after introducing the measures in the following paragraph.

**TABLE 1:** Summary of outcomes presented with mathematical formula; notation defined in footnote

Outcome type	Outcome	Notation
Continuous “individual”	Final volume	${Y_{F}}_{i}$
	Final difference	$\Delta_{i}={Y_{F}}_{i}-{Y_{S}}_{i}$
	Final ratio	$\frac{{Y_{F}}_{i}}{{Y_{S}}_{i}}$
	AUC (all times)	$\frac{D_{2}-D_{1}}{2}\left({Y_{D_{1}}}_{i}+{Y_{D_{2}}}_{i}\right)+\frac{D_{3}-D_{2}}{2}\left({Y_{D_{2}}}_{i}+{Y_{D_{3}}}_{i}\right)$ + …
	AUC (basic)	$\frac{D_{F}-D_{1}}{2}\left({Y_{F}}_{i}+{Y_{S}}_{i}\right)$
Continuous “relative”	TGII (group‐level)	$\frac{\Delta \left(t\right)}{\Delta \left(c\right)}$
	TGII (random pairs)	$\frac{\Delta_{i}\left(t\right)}{\Delta_{j}\left(c\right)}$
	TGII (matched pairs #1)	$\frac{{∆}_{i}\left(t\right)}{\frac{1}{k}\sum_{j=1}^{k}{∆}_{j}\left(c\right)\;}$
	TGII (matched pairs #2)	$\frac{1}{k}\sum_{j=1}^{k}\frac{{∆}_{i}\left(t\right)}{{∆}_{j}\left(c\right)}$
	TGII (common denominator)	$\frac{{∆}_{i}\left(t\right)}{\frac{1}{n}\sum_{j=1}^{n}{∆}_{j}\left(c\right)\;}$
	Relative difference (group‐level)	$\Delta \left(t\right)-\Delta \left(c\right)$
	Relative difference (random pairs)	$\Delta_{i}\left(t\right)-\Delta_{j}\left(c\right)$
	Relative difference (matched pairs #1)	$\Delta_{i}\left(t\right)-\frac{1}{k}\sum_{j=1}^{k}{∆}_{j}\left(c\right)\;$
	Relative difference (matched pairs #2)	$\frac{1}{k}\sum_{j=1}^{k}\Delta_{i}\left(t\right)-{∆}_{j}\left(c\right)$
	Relative difference (common difference)	$\Delta_{i}\left(t\right)-\frac{1}{n}\sum_{j=1}^{n}{∆}_{j}\left(c\right)$

*Note*: *Y* = tumor volume, $D_{1},D_{2},\ldots ,D_{F}=\text{measurement}\;\mathrm{day}$, $S=D_{1}=\text{start}\;\mathrm{day}$, $F=D_{F}=\text{final}\;\mathrm{day}$, $\Delta =Y_{F}-Y_{S}$, *n* = number of observations per group, *k* = *k*‐nearest neighbors, *i* and *j* = individual observations, *t* = treated group, *c* = control group. The *k*‐nearest neighbors are based on the starting tumor volumes, where the *k* number of control tumors are matched to each treated tumor.

Abbreviations: AUC, area under the curve; TGII, tumor growth inhibition index.

Two “relative” continuous outcomes were used: (1) TGII, ∆t∆c, and (2) relative difference, $∆\left(t\right)-∆\left(c\right).\;$; where Δ is the difference in the volumes between the final and the initial tumor measurement, t is the treatment group, and c is the control group. We proposed and evaluated 5 novel estimators each for both TGII and relative difference (Table 1). Four of these novel estimators are applied directly to each treatment group tumor, whereas one is a group‐level estimator. The group‐level estimator is calculated by estimating the individual counterparts separately. The other 4 estimators are identified on the basis of how treatment and control mice are compared for inference based on (1) random pairs, (2) matched pairs #1, (3) matched pairs #2, and (4) common denominator/difference. The random pairs estimator (1) is calculated by randomly pairing each treatment group tumor with a control group tumor given that a PDX study utilizes baseline tumor sizes that are approximately equal in each mouse. The matched pairs estimators (2 and 3) use 2 different variants of the *k*‐nearest‐neighbors approach to match treatment and control tumors together on the basis of their initial tumor volume to better account for potential heterogeneity (Table 1). The common denominator (TGII) or common difference (relative difference) estimator (4) compares each treatment group tumors with the average volume of the control group tumors.

Figure 1 presents example calculations of the 5 “individual” outcomes for a single case. Figure 1A presents the 10 measurements over 30 days for the case. Figure 1B highlights how the final volume, difference, and ratio calculations consider only the initial and final points in their calculations. Figure 1C provides the estimated AUC (all times), which uses all timepoints with an example calculation for one trapezoid between time D_4_ and D_5_. Figure 1D demonstrates the AUC (basic) calculation, which is simplified to ignore the data collected between start and end and, in this case, overestimates the AUC. The methods presented in Figure 1B–D would need to be repeated for each case included in the study.

Figure 2 presents how to calculate the relative difference and TGII for a selection of “relative” approaches. Figure 2A introduces the individual‐level data to be used in the example with 3 treatment cases and 3 control cases. Figure 2B demonstrates the calculations for the group‐level outcomes that take the average volume within each group at study start and finish using all 3 observations within each group. Figure 2C displays how to implement the matched pairs #1 approach with the 2 nearest neighbors for the treatment case with a starting tumor volume of 200 mm^3^. Figure 2D illustrates how the common difference and common denominators are estimated where all control arm data are used to calculate the outcomes, again with respect to the single treatment case with a starting tumor volume of 200 mm^3^. The examples in Figure 2C,D are for only one case, and would need to be reported for each additional case included in the study.

For noncontinuous outcomes, the binary outcome was dichotomized for tumors that grew more than twice their initial volume. Additionally, a 4‐category variable was evaluated following the RECIST criteria.^14^ Lastly, the time‐to‐event outcome uses the number of days until reaching a doubling of tumor volume.

### 2.2 Simulation parameters

The performance of the outcomes was compared using simulated tumor growth over time. Tumor volume was generated form a normal random walk: $Y_{i}=Y_{i-1}+\epsilon_{i}\;$ where $\epsilon_{i}\sim N\left(\mu_{T},\sigma_{T}^{2}\right)\;$ and $Y_{0}\sim N\left(\mu_{S},\sigma_{S}^{2}\right)$ for a trend in tumor growth, T, and the starting tumor volume, S. The simulations were repeated under each combination of the following scenarios: sample size (small or large), signal strength (null, small, or large mean), and amount of noise (small or large variance). All starting volumes were simulated from a common volume, $\mu_{S}$, of 200 mm^3^ with a variance, $\sigma_{S}^{2}$, of 20 mm^3^. Small and large sample sizes were set at 10 and 20 tumors per treatment group, respectively. Control group tumor growth was set at 20 mm^3^ per day (e.g., if initial volume is 200 mm^3^ then final volume after 28 days equals 760 mm^3^). The small and large mean difference in growth rates between groups, $\mu_{T}\left(t\right)-\mu_{T}\left(c\right)$, or signal strength, was set at −5 and − 10 mm^3^, respectively (i.e., treatment group tumor growth was set at 15 mm^3^ and 10 mm^3^ per day). Variances for the growth trends, $\sigma_{T}^{2}$, were set at 1000 and 10 000 for small and large variances, respectively. For the *k*‐nearest neighbor approaches, the closest 5 measures were used. All simulations assume only one tumor per mouse. Data were simulated in R v3.6.0 (Vienna, Austria) across 10 000 simulated trials for each scenario.

### 2.3 Evaluation of outcome performance

Outcomes are evaluated with respect to their power, type‐1 error rate, relative bias of the mean, and relative error of the variance. Relative bias of the mean and relative error of the variance were calculated as the percent difference between the observed and true values, $\left[\frac{\text{observed}-\text{true}}{\text{true}}\right]$ × 100, and is presented as the mean (95% confidence interval [CI]) across the 10 000 simulated trials. The power was calculated as the proportion of iterations with statistically significant results based on the threshold for statistical significance (*α*) of 0.05. Lastly, the null scenarios—where there is no mean difference between treatment and control tumor growth—were simulated to calculate the type‐1 error rate (same calculation as power under the null scenarios).

For statistical testing, the continuous outcomes were compared via univariate linear regression. The “individual” continuous outcomes were compared between treatment groups, but “relative” continuous outcomes used an intercept‐only model, after first subtracting 1 from each “relative” continuous outcome to shift to all outcomes in order to facilitate ease of interpretation since it tests the null value 0 instead of 1 (since, for TGII, the null hypothesis is $\frac{\Delta \left(t\right)}{\Delta \left(c\right)}=1$). The shift transformation was chosen because the log‐transformation is not always mathematically tractable since TGII can be less than zero (i.e., if the tumor size decreases). Both categorical outcomes were tested using either a chi‐squared test or a Fisher's exact test, depending on resulting counts per category. The time‐to‐event outcome was evaluated with a Cox proportional hazards model (using the coxph function from the survival package in R).

### 2.4 Application to real‐world data

The data used to illustrate and implement these approaches examined the effect of the combination of AZD1775 provided by AstraZeneca or purchased from MolPool (Hong Kong) with Navitoclax purchased from Active Biochem (“drug AB”) versus a placebo treatment (“vehicle”). A subset of data from a past experiment on 1 PDX line for triple‐negative breast cancer (TNBC012) is used to illustrate the different methods where 2 tumors were planted into the hind flanks of each mouse as described previously.^2^, ^15^, ^16^ These tumors were repeatedly measured at unequally spaced days. Tumors were excluded in the real‐world data analysis if the initial starting volume was less than 70 mm^3^ or greater than 500 mm^3^. Given the small sample size within each treatment group, a simplifying assumption that all tumors were independent of each other was made for the real‐world data.

The study was carried out in accordance with the National Institutes of Health (NIH) guidelines for the care and use of laboratory animals, and in a facility accredited by the American Association for Accreditation of Laboratory Animal Care. Approval from University of Colorado Animal Care and Use Committee was obtained before the initiation of experiments. All mice were female athymic nude mice.

## 3 RESULTS

### 3.1 Outcome evaluation

We highlight results from the simulation studies to identify the optimal outcomes based on each measure of performance, with Supporting Information presenting all results from the 8 simulation scenarios with respect to bias and variance (Table S1), type‐1 error (Table S2), and power (Table S3). Further, to help visualize the comparisons of the outcomes, histograms of both the relative bias and the relative error of the variance are shown in Figures S1 and S2 (for a single scenario: small sample size, large mean difference, large variance).

Good performance (assessed across all 8 simulation scenarios) for bias and variance was defined as having less than 3% average relative error, with the 95% confidence interval maintaining a narrow width (<10%) while covering 0%. Good performance for the type‐1 error rate was defined as <6%, where ideally the type‐1 error should be 5% (the α level). Good performance for power was subjectively labeled for each scenario on the basis of the results observed across all 18 outcomes. In Table 2, the 5 estimators for each TGII and relative difference were grouped together, and the categorical outcomes were grouped together owing to similar results across each set of outcomes.

**TABLE 2:** General summary of performance across 8 simulated scenarios with good (+) results across all scenarios or poor (−) results

Outcome	Type‐1 error^a^	Power	Bias	Variance
Final volume	+	+	+	+
Final difference	+	+	+	+
Final ratio	+	+	+	−
AUC (all)	+	−	+	−
AUC (basic)	+	+	+	+
TGII^c^	−	−	−	−
Relative difference^c^	−	+	+	+
Categorical	+^b^	−	Not evaluated	Not evaluated
Time to event	+	−	Not evaluated	Not evaluated

^a^ Good (+) type‐1 error rates were defined as less than 6% false positives (i.e., less than 1% inflated). Good power was defined separately for each scenario as being within 10% of the highest observed power, which was set from the outcomes with stable type‐1 error rates. Both good bias and good variance were defined as having less than 3% average relative error, having the 95% confidence interval covering 0% error, and having the confidence interval spanning less than 10%. To be represented as good (+) in this table, the outcome must be “good” in all 8 simulated scenarios. Minus signs (−) denote “poor” results, which was defined as the opposite of good.

^b^ Type‐1 error for the 2 categorical outcomes (binary and the 4‐category RECIST) had type‐1 error rates between 0% and 3% (expecting 5%), which may be a result of extremely poor power for these outcomes.

^c^ Summarized for all 5 estimators (more specific results can be found in Supporting Information Tables S1–S3).

#### 3.1.1 Results for bias

All continuous outcomes except for TGII had good performance (Table 2). The largest bias was found in the scenario with a small sample size, small mean difference, and a large variance, though measures of TGII also had large biases in other scenarios (Table S1). In general, the large‐variance scenario showed more biased results. All 5 TGII estimators were more biased than the other continuous outcomes, where generally the random pairs estimator and the matched pairs #2 estimator were the worst of the TGII outcomes with respect to bias (Table S1). The TGII outcomes were the only outcomes to produce extreme outliers for the relative bias (Figures S1 and S2).

It should be noted that some estimators are equivalent for the estimation of the mean difference, as expected. Namely, the biases were equivalent for the following pairs: (1) the group‐level TGII and the common denominator TGII; (2) the final difference and 3 of the relative difference estimators (group‐level, random pairs, and common difference); (3) both matched pairs for relative difference.

#### 3.1.2 Results for variance

For “individual” continuous outcomes, the final volume, final difference, and AUC (basic) outcomes consistently had the lowest relative error for variance, with less than 1% relative error from the true variance on average for all scenarios. The AUC outcome using all timepoints had an inflated variance across all scenarios (overestimated by 4%–5% on average). The final ratio outcome underestimated the true variance in small‐variance scenarios but overestimated the true variance in large‐variance scenarios.

The relative difference outcomes that did not use the *k*‐nearest‐neighbors algorithm (group‐level, random pairs, and common difference) are not equivalent for variance calculations—as opposed to the bias results—and all resulted in less than 1% relative error from the true variance on average for all scenarios. Conversely, the 2 matched pairs estimators for relative difference have equivalent variance calculations, and these 2 estimators overestimated the variance for both small sample sizes (>4%) and large sample sizes (>9%).

All 5 TGII estimators have unique results for variance, and all overestimated the variance on average. Notably, the most unstable estimates for the variance came from the random pairs and the matched pairs #2 TGII estimators. The matched pairs #1 TGII estimator was unstable for large variances, but only moderately overestimated for small variances (by 8%–13% on average, Table S1). Similarly, the group‐level TGII estimator was generally unstable for large variances, but only overestimated for small variances by 3%–6% on average (Table S1). The best‐performing TGII estimator for variance estimation was the common denominator, where it was also generally unstable for large variances, but only overestimated for small variances by 1%–3% on average (Table S1). Both the group‐level and common denominator TGII estimators had narrower CIs and fewer occurrences where the variance estimate was >1000% error (Table S1).

#### 3.1.3 Results for type‐1 error

Stable type‐1 error rates were labeled as such for simulation results with <6% false positives across all scenarios. All “individual” continuous outcomes and the time‐to‐event outcome had stable type‐1 error rates. Both categorical outcomes had very low type‐1 error rates (some scenarios close to or equal to 0% and all <3%, Table S2).

For relative difference, both the group‐level and the random pairs estimators had stable, if not improved, type‐1 errors. However, the other 3 estimators for relative difference (matched pairs #1, matched pairs #2, and common difference) had inflated type‐1 error rates near 15% (Table S2). Similarly, for TGII, both the group‐level and random pairs estimators had mostly stable type‐1 errors, although the random pairs results were not as consistent across scenarios (from 4% to 7%, Table S2). The matched pairs #1, matched pairs #2, and common difference TGII estimators had type‐1 error rates 13% or greater. Furthermore, the matched pairs #2 TGII estimator had type‐1 error rates between 23% and 38%.

It should be noted that some estimators have equivalent type‐1 error results. The 2 “relative” estimators that use the control group mean as the relative value (i.e., common denominator and common difference) were equivalent. The 2 *k*‐nearest neighbors relative difference estimators were also equivalent.

#### 3.1.4 Results for power

The results for power are shown in decreasing order for sets of outcomes: (1) those with controlled type‐1 error rates, and (2) those with inflated type‐1 error rates. First, for those with controlled type‐1 errors, the group‐level TGII estimator had the highest power (Table S3), followed by the final volume, final difference, and AUC (basic). The next‐highest power was seen for the final ratio and both the random pairs and group‐level relative difference estimators, followed by the AUC with all timepoints. The worst power for continuous outcomes was observed for the random pairs TGII estimator. The time‐to‐event outcome had less power than all continuous outcomes, but was still higher than the extremely low power observed for the 2 categorical outcomes (Table S3). Second, for those with inflated type‐1 errors, the 2 “relative” estimators that use the control group mean as the relative value (i.e., common denominator and common difference) had the highest power. Both matched pairs estimators for relative difference had the next‐highest power, followed by the matched pairs estimators for TGII.

Similar to the type‐1 error results, it should be noted that some estimators are equivalent for the power results. The 2 “relative” estimators that use the reference group mean as the relative value (i.e., common denominator and common difference) were equivalent. The 2 *k*‐nearest neighbors relative difference estimators were equivalent.

### 3.2 Application to real‐world data

**FIGURE 3:**
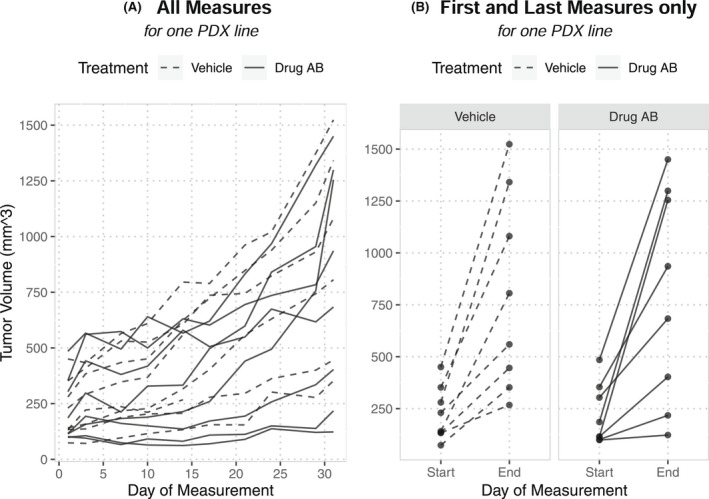
The tumor volumes of the 8 tumors in each treatment group. (A) All measurements taken across the span of the study. (B) Only the first and last measurements for each tumor

Only one PDX line was used (Figure 3) where there were 8 tumors included for each treatment group. The starting volume (mm^3^) for tumors in the “vehicle” group was 74, 133, 134, 141, 230, 280, 353, and 451; the volumes in “drug AB” were 99, 101, 115, 115, 186, 304, 354, and 485. All 18 outcomes were tested for significance with this dataset (Table 3). The results reflect a null study for all continuous and categorical outcomes, where none was statistically significant (i.e., *p* < .05). Results generally reflected these null findings with little to no difference between the 2 groups (Table 3). Both categorical outcomes (binary and the 4 categories of RECIST) did not distinguish between treatment groups because all but one “drug AB” tumor at least doubled in size compared with their original volume. The time‐to‐event outcome did produce a significant result (*p* = .010), indicating that the treatment was better than the control with respect to the time until the tumor doubled, where the median days to tumor doubling was 15.5 days for the control group and 24 days for the treated group. These results help to illustrate the practical challenges of selecting outcomes, such as having most data in a single categorical group, and the information ultimately contained in the summary, such as the time to tumor doubling versus just considering volume absent time.

**TABLE 3:** Results from univariate testing for all 18 outcomes on real‐world data

Outcome	Estimate^a^	*p*‐Value
Final volume	−1.30	.996
Final difference	3.29	.987
Final ratio	0.40	.752
AUC (all times)	−353.18	.932
AUC (basic)	1176.58	.810
TGII (group‐level)	1.01	.988
TGII (random pairs)	0.54	.359
TGII (matched pairs #1)	0.16	.691
TGII (matched pairs #2)	0.56	.337
TGII (common denominator)	0.01	.984
Relative difference (group‐level)	3.29	.988
Relative difference (random pairs)	3.29	.987
Relative difference (matched pairs #1)	52.28	.748
Relative difference (matched pairs #2)	52.28	.748
Relative difference (common difference)	3.29	.984
Binary	–	1.000
Categorical (RECIST)	–	1.000
Time to event of tumor doubling	0.12	.010

^a^ First, the 5 estimates for the “individual” continuous outcomes are mean differences between treatment groups, where a positive estimate represents larger tumors in the treated group compared with the control. Second, the 5 estimates for TGII directly represent the estimand of interest $\left(\frac{\Delta \left(t\right)}{\Delta \left(c\right)}\right)$; values greater than 1 represent larger tumor growth in the treated group compared with the control. Third, the 5 estimates for relative difference are mean differences between treatment groups, which is also the estimand of interest ($\Delta \left(t\right)-\Delta \left(c\right)$); positive values represent larger tumor growth in the treated group compared with the control. Fourth, estimates are not provided for the categorical outcomes because all tumors had the same fate for both treatment groups. Lastly, the estimate for the time‐to‐event outcome is a hazard ratio for the treatment group compared with the control group; a value less than 1 represents a lower hazard of the tumor doubling compared with the control.

## 4 DISCUSSION

The simulation results and data application provide evidence that certain outcomes perform better with respect to statistical considerations such as the bias of the estimator, its variance, and the ability to detect a difference (power) or lack thereof (type I error). If one outcome is to be selected, we would recommend prioritizing individual‐level measures of the final volume or difference. Alternatively, the use of a time‐to‐event measure may be advantageous given its performance in the simulation studies and the ability to estimate the median or mean time until an event occurs within each group. However, since follow‐up measurements are set at fixed intervals, the day that a tumor reaches a certain threshold may not actually be observed and reflect interval censoring. Thus, the time‐to‐event outcome may be an overestimate of the true time until the event, for example, when the tumor has the event at day 5 but it is not observed until its follow‐up measurement on day 7.

Generally, TGII is not recommended for use as a statistical outcome given its poor performance across these measures. However, if there is a strong desire to use TGII as the outcome, then the common denominator estimator (or the group‐level estimator since these 2 are equivalent for estimation of the mean) would be the best option. Additionally, the 2 categorical outcomes are further scrutinized because of the limited interpretation that these provide and the loss of information when using such outcomes. Sharma, Maitland, and Ratain previously discussed that using the RECIST categories as the primary outcome is inadequate in many scenarios.^17^

When considering unplanned missing data, the most common source comes from euthanized mice, which poses a problem for the more straightforward univariate analyses. Unfortunately, the common approach used in cross‐sectional studies of imputing with the last observation carried forward (LOCF) is problematic.^18^ Fortunately, we observe the cause of the missing data (i.e., we have the previous volume that crossed the threshold to euthanize), and there are analysis methods that adequately handle this type of missingness in a multivariable regression framework with maximum‐likelihood‐based methods.^19^

The application to the real‐world data demonstrated similar null statistical conclusions for most outcomes except for the time to event of tumor doubling. However, it would be naïve to assume that all outcomes also have the same interpretation or implications, where the time to event addresses a different underlying question of interest than raw tumor size or relative change in tumor size. If timing is a crucial aspect of the study, then methods accounting for the longitudinal nature of the data and the research question of interest should be used.

There are limitations to consider. First, our simulations used only linear growth trends, where other nonlinear trends may be more realistic. However, many analytical approaches propose transforming non‐normal data first to meet modeling assumptions. Second, tumor volumes have a lower limit (i.e., volumes cannot be less than zero), yet we assume that the outcome is normally distributed without a lower cutoff in the simulations. However, it is encouraging that the analogous results from other simulations with a lower truncation (not included) did draw different conclusions. In practice, there are other viable approaches that would account for the lower truncation, such as a mixture model in a multivariate regression framework. Third, not all 18 proposed outcomes are entirely unique. For example, the final difference is essentially the same outcome as the group‐level relative difference estimator, but they were utilized in different testing frameworks. Another example worth noting is that the TGII versus relative difference estimand are very similar, where the null hypothesis is algebraically the same, but the outcomes follow different distributions. Fourth, we acknowledge the limitation of using simple summary statistics (e.g., means) for the TGII results, since we observed extreme outliers. Fifth, the outcomes tested here are not an exhaustive list of all relevant outcomes, nor is this an exhaustive list of all relevant ways to use these outcomes. Furthermore, certain data transformations may be justified, such as the log‐transformation (especially with the final ratio where log(*x*/*y*) becomes log(*x*) − log(*y*)). Also, certain situations may also render some outcomes useless for comparison, such as the binary outcome of “doubling in size,” if in fact all tumors doubled in size.

A final limitation worth highlighting stems from studies that use multiple PDX lines but summarize the results within each PDX line separately. While not directly addressed in this paper, it is possible to generalize our results across multiple PDX lines by using statistical methods that can account for the relevant potential sources of correlation (e.g., spatial, temporal, hierarchical). In other words, one might see 2 options for analyzing data with multiple PDX lines: (1) less efficiently, by repeating the same analysis for each PDX line, or (2) more efficiently, by using a single mixed‐effect regression model, which could appropriately model the complex correlation within and across PDX lines in a single overarching study. More specifically, we recommend modeling the correlation in tumor growth with a Kronecker product correlation structure to account for the repeated measures over time and the spatial correlation of the 2 tumors in a single mouse, all nested in a random intercept for each PDX line, provided that the feasibility of fitting complex statistical models in not a concern. This is similarly reflected in the results of Oberg et al., who explored the use of linear mixed‐effects models with complex correlation structures in the context of ovarian cancer PDX studies.^20^ Additional work has examined the need for unified frameworks of PDX studies and their evaluation to better ensure replicability and optimal use of study data.^5^, ^21^, ^22^

## 5 CONCLUSION

Final volume and final difference showed encouraging performance across all 4 properties evaluated. The only notable shortcoming for the AUC (basic) outcome was the decreased power is some scenarios. The AUC (all timepoints) outcome had inflated variance in all scenarios explored, leading to decreased power. The final ratio performed much better than the other ratio‐based outcomes (i.e., TGII), but still had decreased power and unstable variance estimates. None of the TGII estimators performed as well with respect to statistical properties as the more straightforward final volume or final difference for any of the evaluations in any of the scenarios and were also prone to extreme under or overestimates for both bias and variance. This suggests that, while TGII estimators may be useful as a descriptive summary, other measures may have better properties for statistical analysis with respect to power, type I error, and bias. Although the relative difference estimators were unbiased, some variations had inflated type‐1 error rates. The 2 categorical outcomes have substantially lower power than all other outcomes. The time‐to‐event outcome has inherent measurement error (biased time to event due to the timing of measurements) and decreased power. If a time‐to‐event outcome is desired, then the study design should consider measuring the tumor volumes on all days—rather than at fixed intervals—to eliminate the measurement bias introduced by interval censoring. Furthermore, if the timing of the study is of interest, then other time‐varying regression methods should also be considered.

Outcome selection is partially a subjective choice for evaluating PDX studies, which leads to challenges in the consistency of reporting findings in this field. The approach using “relative” continuous outcomes, such as the TGII, is inferior to using “individual” continuous outcomes, such as the raw tumor volume, based on the simulation results presented in this paper. Our results suggest that some of the more straightforward, intuitive outcomes are the most consistent and powerful, while more complex measures or the proposed TGII summaries are more variable and perform poorly. While these simulations considered only the case with 2 groups within one PDX line, the results and methods are generalizable to cases with multiple treatments and multiple PDX lines. It is also worth noting that these results are not exclusive to PDX studies and may be applied to other contexts, such as for studies of xenografting cancer cell lines. The simulation results give empirical reasoning to specify the outcome as either the final tumor volume or the change in volume from baseline. Fortunately, these outcomes work well in a multivariate regression model, which may be the most efficient analysis approach for PDX studies.

## AUTHOR CONTRIBUTIONS

LP and AK made substantial contributions to the conception and design of the work. All co‐authors contributed to the drafting of the work and approval for final submission.

## FUNDING INFORMATION

Alexander Kaizer was supported by NHLBI K01‐HL151754

## CONFLICT OF INTEREST

The co‐authors have no conflicts to disclose.

## Supporting information


Table S1‐S3
