# Supplementary material for: Assessing the performance of different outcomes for tumor growth studies with animal models
Source: Animal Model Exp Med. 2022 Jun 14;5(3):248–57. doi: 10.1002/ame2.12250 (PMC9240739; doi:10.1002/ame2.12250)
Supplement: Supplementary file 1 — Table S1‐S3 [file AME2-5-248-s001.docx]

**SUPPLEMENTARY MATERIALS for “Assessing the Performance of Different Outcomes for Tumor Growth Studies with Animal Models”**

Luke W Patten, Patrick Blatchford, Matthew Strand, and Alexander M Kaizer

Table S1. Bias and variance results. Superscripts represent the number of simulations (out of 10000) that resulted in greater than 1000% error. n=sample size, $\sigma^{2}$=variance, $\mu$=mean difference.

| **Outcome** | **n** | $\boldsymbol{\sigma}^{\boldsymbol{2}}$ | $\boldsymbol{\mu}$ | **Relative Bias (%)** | **95% Confidence Interval** | **Relative Error for Variance (%)** | **95% Confidence**  **Interval** |
| --- | --- | --- | --- | --- | --- | --- | --- |
| **Final Volume** | Small | Small | Small | -0.65 | (-1.69, 0.40) | -0.15 | (-0.81, 0.51) |
|  |  |  | Large | -0.25 | (-0.78, 0.27) | 0.17 | (-0.48, 0.82) |
|  |  | Large | Small | -2.15 | (-5.45, 1.15) | 0.37 | (-0.28, 1.03) |
|  |  |  | Large | -0.49 | (-2.15, 1.17) | -0.17 | (-0.82, 0.48) |
|  | Large | Small | Small | -0.08 | (-0.82, 0.66) | -0.13 | (-0.58, 0.32) |
|  |  |  | Large | 0.03 | (-0.34, 0.41) | 0.09 | (-0.35, 0.54) |
|  |  | Large | Small | -0.56 | (-2.90, 1.79) | -0.45 | (-0.90, 0.00) |
|  |  |  | Large | 0.86 | (-0.30, 2.02) | 0.38 | (-0.07, 0.82) |
| **Final Difference** | Small | Small | Small | -0.64 | (-1.68, 0.40) | -0.16 | (-0.82, 0.49) |
|  |  |  | Large | -0.29 | (-0.82, 0.23) | 0.19 | (-0.47, 0.84) |
|  |  | Large | Small | -2.24 | (-5.54, 1.06) | 0.41 | (-0.24, 1.07) |
|  |  |  | Large | -0.50 | (-2.16, 1.16) | -0.15 | (-0.79, 0.50) |
|  | Large | Small | Small | -0.08 | (-0.82, 0.65) | -0.14 | (-0.58, 0.31) |
|  |  |  | Large | 0.04 | (-0.33, 0.41) | 0.15 | (-0.29, 0.60) |
|  |  | Large | Small | -0.66 | (-3.01, 1.68) | -0.43 | (-0.88, 0.02) |
|  |  |  | Large | 0.89 | (-0.27, 2.05) | 0.38 | (-0.07, 0.83) |
| **Final Ratio** | Small | Small | Small | -0.61 | (-1.71, 0.49) | -9.36 | (-9.98, -8.74) |
|  |  |  | Large | -0.28 | (-0.83, 0.26) | -4.75 | (-5.39, -4.10) |
|  |  | Large | Small | -2.48 | (-5.82, 0.87) | 2.31 | (1.62, 3.00) |
|  |  |  | Large | -0.56 | (-2.24, 1.11) | 2.04 | (1.36, 2.72) |
|  | Large | Small | Small | -0.14 | (-0.91, 0.63) | -9.28 | (-9.70, -8.85) |
|  |  |  | Large | 0.06 | (-0.32, 0.45) | -4.76 | (-5.21, -4.32) |
|  |  | Large | Small | -0.87 | (-3.24, 1.49) | 1.30 | (0.83, 1.77) |
|  |  |  | Large | 0.91 | (-0.26, 2.08) | 2.54 | (2.06, 3.01) |
| **AUC** *(all)*  **AUC**  *(all)* | Small | Small | Small | -0.71 | (-1.93, 0.51) | 4.34 | (3.65, 5.02) |
|  |  |  | Large | -0.25 | (-0.86, 0.37) | 4.34 | (3.66, 5.02) |
|  |  | Large | Small | -1.63 | (-5.44, 2.19) | 5.09 | (4.40, 5.78) |
|  |  |  | Large | -0.36 | (-2.28, 1.56) | 4.48 | (3.80, 5.15) |
|  | Large | Small | Small | -0.27 | (-1.14, 0.60) | 3.84 | (3.37, 4.31) |
|  |  |  | Large | 0.33 | (-0.11, 0.77) | 3.82 | (3.36, 4.29) |
|  |  | Large | Small | 0.20 | (-2.50, 2.90) | 3.85 | (3.39, 4.32) |
|  |  |  | Large | 1.06 | (-0.29, 2.40) | 4.61 | (4.14, 5.08) |
| **AUC**  *(basic)* | Small | Small | Small | -0.65 | (-1.72, 0.41) | -0.12 | (-0.78, 0.53) |
|  |  |  | Large | -0.21 | (-0.75, 0.32) | 0.16 | (-0.50, 0.81) |
|  |  | Large | Small | -2.05 | (-5.36, 1.25) | 0.33 | (-0.32, 0.99) |
|  |  |  | Large | -0.48 | (-2.14, 1.18) | -0.19 | (-0.84, 0.45) |
|  | Large | Small | Small | -0.08 | (-0.83, 0.68) | -0.12 | (-0.57, 0.32) |
|  |  |  | Large | 0.03 | (-0.36, 0.41) | 0.03 | (-0.42, 0.48) |
|  |  | Large | Small | -0.45 | (-2.80, 1.90) | -0.47 | (-0.92, -0.03) |
|  |  |  | Large | 0.83 | (-0.33, 1.99) | 0.37 | (-0.08, 0.82) |
| **TGII**  *(group-level)* | Small | Small | Small | 1.09 | (0.77, 1.40) | 6.41 | (5.43, 7.39) |
|  |  |  | Large | 1.12 | (0.70, 1.54) | 5.44 | (4.47, 6.41) |
|  |  | Large | Small | 18.21 | (13.45, 22.96) | 5458158.88 ^410^ | (-5146383.38, 16062701.14) |
|  |  |  | Large | 14.74 | (12.25, 17.24) | 25956.17 ^312^ | (-6730.49, 58642.82) |
|  | Large | Small | Small | 0.46 | (0.24, 0.68) | 2.72 | (2.09, 3.36) |
|  |  |  | Large | 0.41 | (0.12, 0.71) | 2.76 | (2.11, 3.42) |
|  |  | Large | Small | 4.47 | (1.92, 7.03) | 581403.75 ^87^ | (-558092.90, 1720900.41) |
|  |  |  | Large | 4.28 | (3.26, 5.30) | 36.02 ^28^ | (32.42, 39.62) |
| **TGII**  *(random pairs)*  **TGII**  *(random pairs)* | Small | Small | Small | 13.30 | (8.83, 17.77) | 20883.82 ^312^ | (-4823.95, 46591.60) |
|  |  |  | Large | 14.92 | (11.46, 18.39) | 6898.78 ^253^ | (572.46, 13225.11) |
|  |  | Large | Small | -154.89 | (-388.16, 78.39) | 5707229.82 ^3687^ | (-2350189.46, 13764649.11) |
|  |  |  | Large | -232.00 | (-527.04, 63.04) | 5076777.07 ^2523^ | (-1530893.69, 11684447.83) |
|  | Large | Small | Small | 11.67 | (7.99, 15.34) | 28195.37 ^400^ | (-7101.02, 63491.76) |
|  |  |  | Large | 12.93 | (11.28, 14.58) | 3060.85 ^268^ | (-385.78, 6507.48) |
|  |  | Large | Small | -80.38 | (-217.67, 56.92) | 3961642.34 ^4920^ | (-540401.11, 8463685.79) |
|  |  |  | Large | 68.50 | (-87.72, 224.72) | 2843851.07 ^4787^ | (359792.51, 5327909.64) |
| **TGII**  *(matched*  *pairs #1)* | Small | Small | Small | 2.00 | (1.67, 2.34) | 9.21 | (8.03, 10.40) |
|  |  |  | Large | 2.10 | (1.66, 2.54) | 7.56 | (6.42, 8.71) |
|  |  | Large | Small | 44.89 | (-5.59, 95.36) | 155738.15 ^800^ | (-448.36, 311924.65) |
|  |  |  | Large | 58.53 | (12.49, 104.57) | 81110.70 ^703^ | (4561.18, 157660.23) |
|  | Large | Small | Small | 1.96 | (1.72, 2.20) | 12.84 | (11.94, 13.73) |
|  |  |  | Large | 1.79 | (1.47, 2.10) | 9.48 | (8.64, 10.31) |
|  |  | Large | Small | 39.74 | (-2.63, 82.12) | 305963.25 ^1142^ | (60602.59, 551323.91) |
|  |  |  | Large | 70.12 | (-15.17, 155.41) | 1339771.94 ^1071^ | (-423819.16, 3103363.04) |
| **TGII**  *(matched*  *pairs #2)* | Small | Small | Small | 15.33 | (11.84, 18.81) | 1073.34 ^101^ | (207.20, 1939.47) |
|  |  |  | Large | 14.28 | (11.50, 17.06) | 1023.38 ^75^ | (265.70, 1781.07) |
|  |  | Large | Small | -14.66 | (-83.60, 54.27) | 224718.49 ^2155^ | (23734.64, 425702.35) |
|  |  |  | Large | -156.44 | (-375.07, 62.19) | 1560509.07 ^1959^ | (-278916.96, 3399935.11) |
|  | Large | Small | Small | 12.02 | (7.05, 16.99) | 7791.78 ^175^ | (-346.77, 15930.34) |
|  |  |  | Large | 13.49 | (11.92, 15.06) | 545.45 ^103^ | (75.20, 1015.69) |
|  |  | Large | Small | -16.01 | (-102.84, 70.83) | 617828.82 ^3146^ | (56722.02, 1178935.63) |
|  |  |  | Large | 143.97 | (-108.40, 396.33) | 2192206.83 ^2835^ | (-838884.56, 5223298.22) |
| **TGII**  *(common denominator)* | Small | Small | Small | 1.09 | (0.77, 1.40) | 3.15 | (2.10, 4.20) |
|  |  |  | Large | 1.12 | (0.70, 1.54) | 3.26 | (2.21, 4.31) |
|  |  | Large | Small | 18.21 | (13.45, 22.96) | 508.42 ^133^ | (-124.35, 1141.18) |
|  |  |  | Large | 14.74 | (12.25, 17.24) | 119.56 ^108^ | (68.90, 170.22) |
|  | Large | Small | Small | 0.46 | (0.24, 0.68) | 1.16 | (0.45, 1.87) |
|  |  |  | Large | 0.41 | (0.12, 0.71) | 1.81 | (1.09, 2.52) |
|  |  | Large | Small | 4.47 | (1.92, 7.03) | 203.62 ^10^ | (-158.33, 565.57) |
|  |  |  | Large | 4.28 | (3.26, 5.30) | 16.45 ^3^ | (14.89, 18.02) |
| **Difference**  *(group-level)*  **Difference**  *(group-level)* | Small | Small | Small | -0.64 | (-1.68, 0.40) | -0.16 | (-0.82, 0.49) |
|  |  |  | Large | -0.29 | (-0.82, 0.23) | 0.19 | (-0.47, 0.84) |
|  |  | Large | Small | -2.24 | (-5.54, 1.06) | 0.41 | (-0.24, 1.07) |
|  |  |  | Large | -0.50 | (-2.16, 1.16) | -0.15 | (-0.79, 0.50) |
|  | Large | Small | Small | -0.08 | (-0.82, 0.65) | -0.14 | (-0.58, 0.31) |
|  |  |  | Large | 0.04 | (-0.33, 0.41) | 0.15 | (-0.29, 0.60) |
|  |  | Large | Small | -0.66 | (-3.01, 1.68) | -0.43 | (-0.88, 0.02) |
|  |  |  | Large | 0.89 | (-0.27, 2.05) | 0.38 | (-0.07, 0.83) |
| **Difference**  *(random pairs)* | Small | Small | Small | -0.64 | (-1.68, 0.40) | -0.59 | (-1.52, 0.33) |
|  |  |  | Large | -0.29 | (-0.82, 0.23) | 0.29 | (-0.64, 1.22) |
|  |  | Large | Small | -2.24 | (-5.54, 1.06) | 0.03 | (-0.91, 0.97) |
|  |  |  | Large | -0.50 | (-2.16, 1.16) | 0.16 | (-0.77, 1.08) |
|  | Large | Small | Small | -0.08 | (-0.82, 0.65) | -0.09 | (-0.74, 0.55) |
|  |  |  | Large | 0.04 | (-0.33, 0.41) | -0.07 | (-0.70, 0.56) |
|  |  | Large | Small | -0.66 | (-3.01, 1.68) | -0.64 | (-1.27, -0.01) |
|  |  |  | Large | 0.89 | (-0.27, 2.05) | 0.69 | (0.06, 1.33) |
| **Difference**  *(matched*  *pairs #1)* | Small | Small | Small | -0.55 | (-1.64, 0.55) | 4.02 | (3.05, 5.00) |
|  |  |  | Large | -0.40 | (-0.95, 0.15) | 4.00 | (3.03, 4.96) |
|  |  | Large | Small | -2.63 | (-6.14, 0.88) | 4.46 | (3.49, 5.43) |
|  |  |  | Large | -0.57 | (-2.33, 1.19) | 4.11 | (3.13, 5.09) |
|  | Large | Small | Small | -0.24 | (-1.03, 0.54) | 9.80 | (9.07, 10.53) |
|  |  |  | Large | 0.20 | (-0.20, 0.60) | 10.37 | (9.64, 11.09) |
|  |  | Large | Small | -1.16 | (-3.67, 1.36) | 9.64 | (8.92, 10.35) |
|  |  |  | Large | 0.74 | (-0.51, 1.98) | 9.71 | (8.99, 10.44) |
| **Difference**  *(matched*  *pairs #2)* | Small | Small | Small | -0.55 | (-1.64, 0.55) | 4.02 | (3.05, 5.00) |
|  |  |  | Large | -0.40 | (-0.95, 0.15) | 4.00 | (3.03, 4.96) |
|  |  | Large | Small | -2.63 | (-6.14, 0.88) | 4.46 | (3.49, 5.43) |
|  |  |  | Large | -0.57 | (-2.33, 1.19) | 4.11 | (3.13, 5.09) |
|  | Large | Small | Small | -0.24 | (-1.03, 0.54) | 9.80 | (9.07, 10.53) |
|  |  |  | Large | 0.20 | (-0.20, 0.60) | 10.37 | (9.64, 11.09) |
|  |  | Large | Small | -1.16 | (-3.67, 1.36) | 9.64 | (8.92, 10.35) |
|  |  |  | Large | 0.74 | (-0.51, 1.98) | 9.71 | (8.99, 10.44) |
| **Difference** *(common difference)* | Small | Small | Small | -0.64 | (-1.68, 0.40) | 0.11 | (-0.82, 1.03) |
|  |  |  | Large | -0.29 | (-0.82, 0.23) | 0.31 | (-0.61, 1.24) |
|  |  | Large | Small | -2.24 | (-5.54, 1.06) | 0.48 | (-0.44, 1.41) |
|  |  |  | Large | -0.50 | (-2.16, 1.16) | -0.09 | (-1.01, 0.82) |
|  | Large | Small | Small | -0.08 | (-0.82, 0.65) | -0.29 | (-0.93, 0.35) |
|  |  |  | Large | 0.04 | (-0.33, 0.41) | 0.35 | (-0.29, 1.00) |
|  |  | Large | Small | -0.66 | (-3.01, 1.68) | -0.15 | (-0.78, 0.49) |
|  |  |  | Large | 0.89 | (-0.27, 2.05) | -0.07 | (-0.71, 0.57) |

Table S2. Type-1 error for all scenarios. n=sample size, $\sigma^{2}$=variance.

| **Outcome** | **Type-1 Error** | | | |
| --- | --- | --- | --- | --- |
|  | **Small n** | | **Large n** | |
|  | **Small** $\boldsymbol{\sigma}^{\boldsymbol{2}}$ | **Large** $\boldsymbol{\sigma}^{\boldsymbol{2}}$ | **Small** $\boldsymbol{\sigma}^{\boldsymbol{2}}$ | **Large** $\boldsymbol{\sigma}^{\boldsymbol{2}}$ |
| **Final Volume** | 0.0488 | 0.0490 | 0.0519 | 0.0495 |
| **Final Difference** | 0.0489 | 0.0500 | 0.0513 | 0.0494 |
| **Final Ratio** | 0.0496 | 0.0511 | 0.0498 | 0.0492 |
| **AUC**  *(all times)* | 0.0479 | 0.0489 | 0.0528 | 0.0510 |
| **AUC**  *(basic)* | 0.0468 | 0.0486 | 0.0538 | 0.0494 |
| **TGII**  *(group-level)* | 0.0356 | 0.0489 | 0.0437 | 0.0474 |
| **TGII**  *(random pairs)* | 0.0434 | 0.0393 | 0.0712 | 0.0382 |
| **TGII**  *(matched pairs #1)* | 0.1477 | 0.1299 | 0.1587 | 0.1321 |
| **TGII**  (*matched pairs #2)* | 0.2339 | 0.3702 | 0.3246 | 0.3762 |
| **TGII**  *(common denominator)* | 0.1409 | 0.1420 | 0.1586 | 0.1526 |
| **Relative Difference** *(group-level)* | 0.0351 | 0.0351 | 0.0450 | 0.0435 |
| **Relative Difference** *(random pairs)* | 0.0489 | 0.0476 | 0.0508 | 0.0472 |
| **Relative Difference** *(matched pairs #1)* | 0.1472 | 0.1533 | 0.1556 | 0.1553 |
| **Relative Difference** *(matched pairs #2)* | 0.1472 | 0.1533 | 0.1556 | 0.1553 |
| **Relative Difference** *(common difference)* | 0.1409 | 0.1420 | 0.1586 | 0.1526 |
| **Binary** | 0.0000 | 0.0102 | 0.0001 | 0.0229 |
| **Categorical**  *(RECIST)* | 0.0000 | 0.0072 | 0.0000 | 0.0280 |
| **Time-to-event** | 0.0450 | 0.0496 | 0.0506 | 0.0529 |

Table S3. Power for all scenarios. n=sample size, $\sigma^{2}$=variance, $\mu$=mean difference.

| **Outcome of Interest** | **Power** | | | | | | | |
| --- | --- | --- | --- | --- | --- | --- | --- | --- |
|  | **Small n** | | | | **Large n** | | | |
|  | **Small** $\boldsymbol{\sigma}^{\boldsymbol{2}}$ | | **Large** $\boldsymbol{\sigma}^{\boldsymbol{2}}$ | | **Small** $\boldsymbol{\sigma}^{\boldsymbol{2}}$ | | **Large** $\boldsymbol{\sigma}^{\boldsymbol{2}}$ | |
|  | **Small** $\boldsymbol{\mu}$ | **Large** $\boldsymbol{\mu}$ | **Small** $\boldsymbol{\mu}$ | **Large** $\boldsymbol{\mu}$ | **Small** $\boldsymbol{\mu}$ | **Large** $\boldsymbol{\mu}$ | **Small** $\boldsymbol{\mu}$ | **Large** $\boldsymbol{\mu}$ |
| **Final Volume** | 0.4200 | 0.9397 | 0.0832 | 0.2025 | 0.7287 | 0.9993 | 0.1244 | 0.3712 |
| **Final Difference** | 0.4290 | 0.9431 | 0.0843 | 0.2023 | 0.7315 | 0.9991 | 0.1246 | 0.3733 |
| **Final Ratio** | 0.4000 | 0.9242 | 0.0824 | 0.1994 | 0.6871 | 0.9983 | 0.1214 | 0.3715 |
| **AUC**  *(all times)* | 0.3162 | 0.8531 | 0.0731 | 0.1644 | 0.5902 | 0.9916 | 0.1121 | 0.2982 |
| **AUC**  *(basic)* | 0.4051 | 0.9320 | 0.0828 | 0.2028 | 0.7095 | 0.9989 | 0.1245 | 0.3695 |
| **TGII**  *(group-level)* | 0.4727 | 0.9589 | 0.1392 | 0.3240 | 0.7692 | 0.9997 | 0.2023 | 0.5159 |
| **TGII**  *(random pairs)* | 0.2880 | 0.7884 | 0.0838 | 0.1652 | 0.3874 | 0.9142 | 0.0910 | 0.1932 |
| **TGII**  *(matched pairs #1)* | 0.5820 | 0.9681 | 0.1804 | 0.3256 | 0.8074 | 0.9994 | 0.1952 | 0.4126 |
| **TGII**  (*matched pairs #2)* | 0.3979 | 0.8678 | 0.3707 | 0.4237 | 0.5268 | 0.9582 | 0.3796 | 0.4409 |
| **TGII**  *(common denominator)* | 0.6172 | 0.9816 | 0.1996 | 0.3708 | 0.8819 | 1.0000 | 0.2846 | 0.5903 |
| **Relative Difference** *(group-level)* | 0.3700 | 0.9217 | 0.0620 | 0.1629 | 0.7115 | 0.9991 | 0.1115 | 0.3492 |
| **Relative Difference** *(random pairs)* | 0.3930 | 0.9122 | 0.0824 | 0.1861 | 0.7110 | 0.9984 | 0.1245 | 0.3581 |
| **Relative Difference** *(matched pairs #1)* | 0.5925 | 0.9719 | 0.2012 | 0.3550 | 0.8392 | 0.9996 | 0.2757 | 0.5532 |
| **Relative Difference** *(matched pairs #2)* | 0.5925 | 0.9719 | 0.2012 | 0.3550 | 0.8392 | 0.9996 | 0.2757 | 0.5532 |
| **Relative Difference** *(common difference)* | 0.6172 | 0.9816 | 0.1996 | 0.3708 | 0.8819 | 1.0000 | 0.2846 | 0.5903 |
| **Binary** | 0.0010 | 0.1594 | 0.0183 | 0.0493 | 0.0278 | 0.7095 | 0.0520 | 0.1450 |
| **Categorical**  *(RECIST)* | 0.0000 | 0.0006 | 0.0164 | 0.0536 | 0.0000 | 0.0148 | 0.0633 | 0.1633 |
| **Time-to-event** | 0.2085 | 0.7191 | 0.0725 | 0.1498 | 0.3988 | 0.9638 | 0.1005 | 0.2662 |

Figure S1. Histograms for the 10000 relative biases (%) from the 10000 simulations under the small sample size, large mean, large variance scenario. **a**.) The x-scales are flexible across the outcomes, where the smallest range was used while including all values for each outcome. **b**.) The limits for the x-scale were determined by the maximum value observed between the Final Volume, Final Difference, and AUC (basic); any values outside of this range is not included.


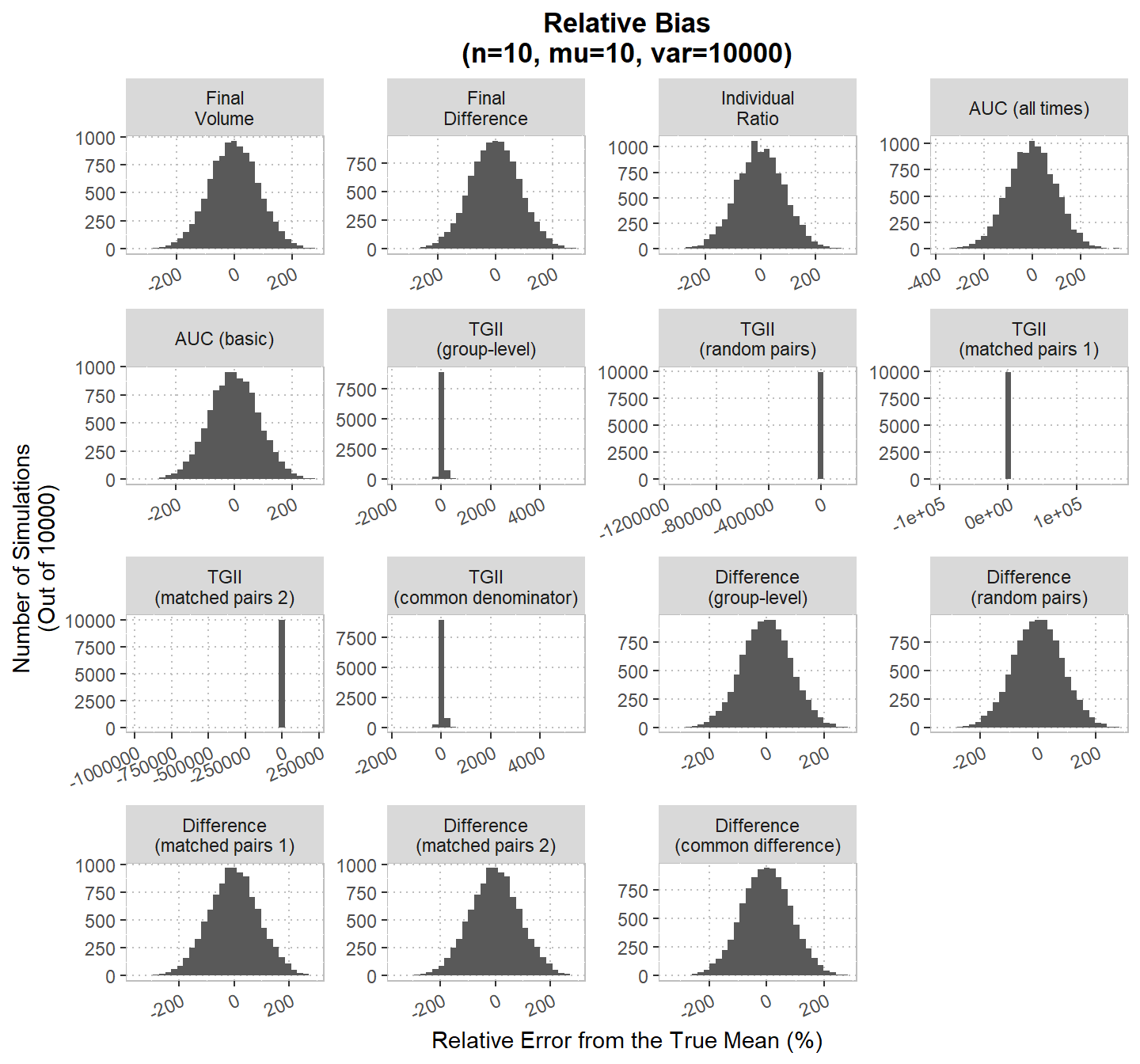


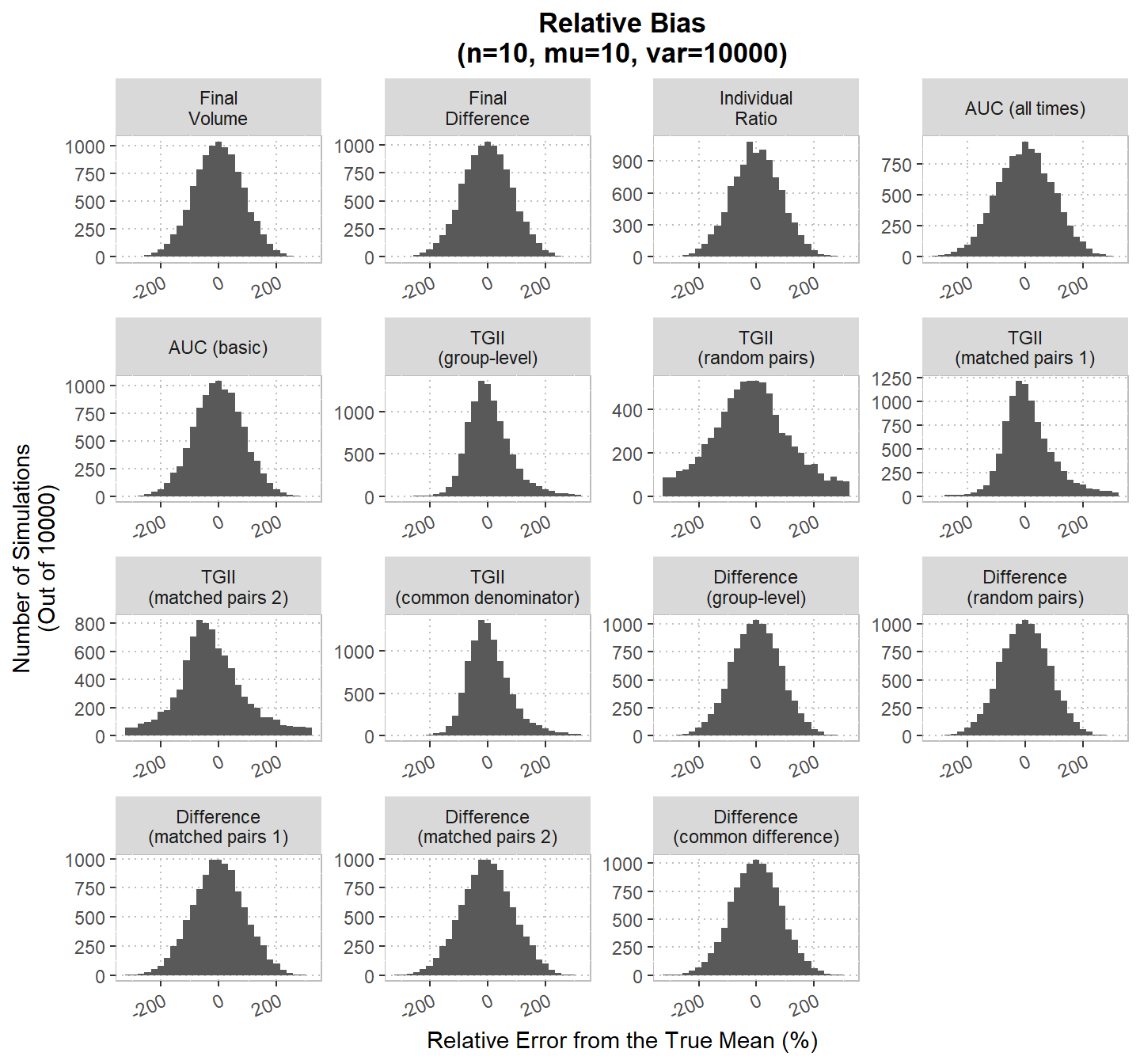


**b.**

**a.**

Figure S2. Histograms for the 10000 relative errors of the variance (%) from all 10000 simulations under the small sample size/large mean/large variance scenario. **a**.) The x-scales are flexible across the outcomes, where the smallest range was used while including all values for each outcome. **b**.) The limits for the x-scale were determined by the maximum value observed between the Final Volume, Final Difference, and AUC (basic); any values outside of this range is not included.


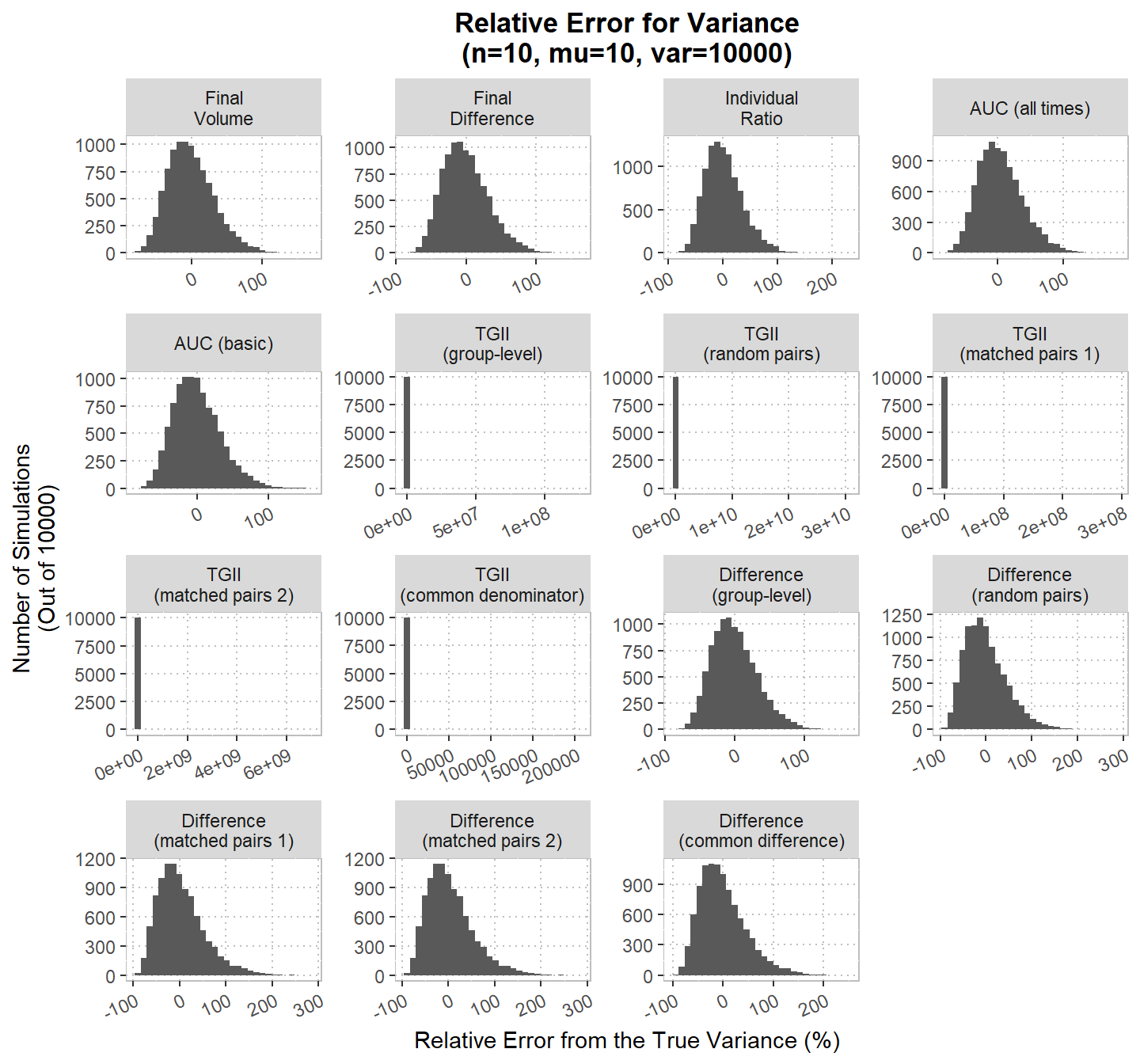

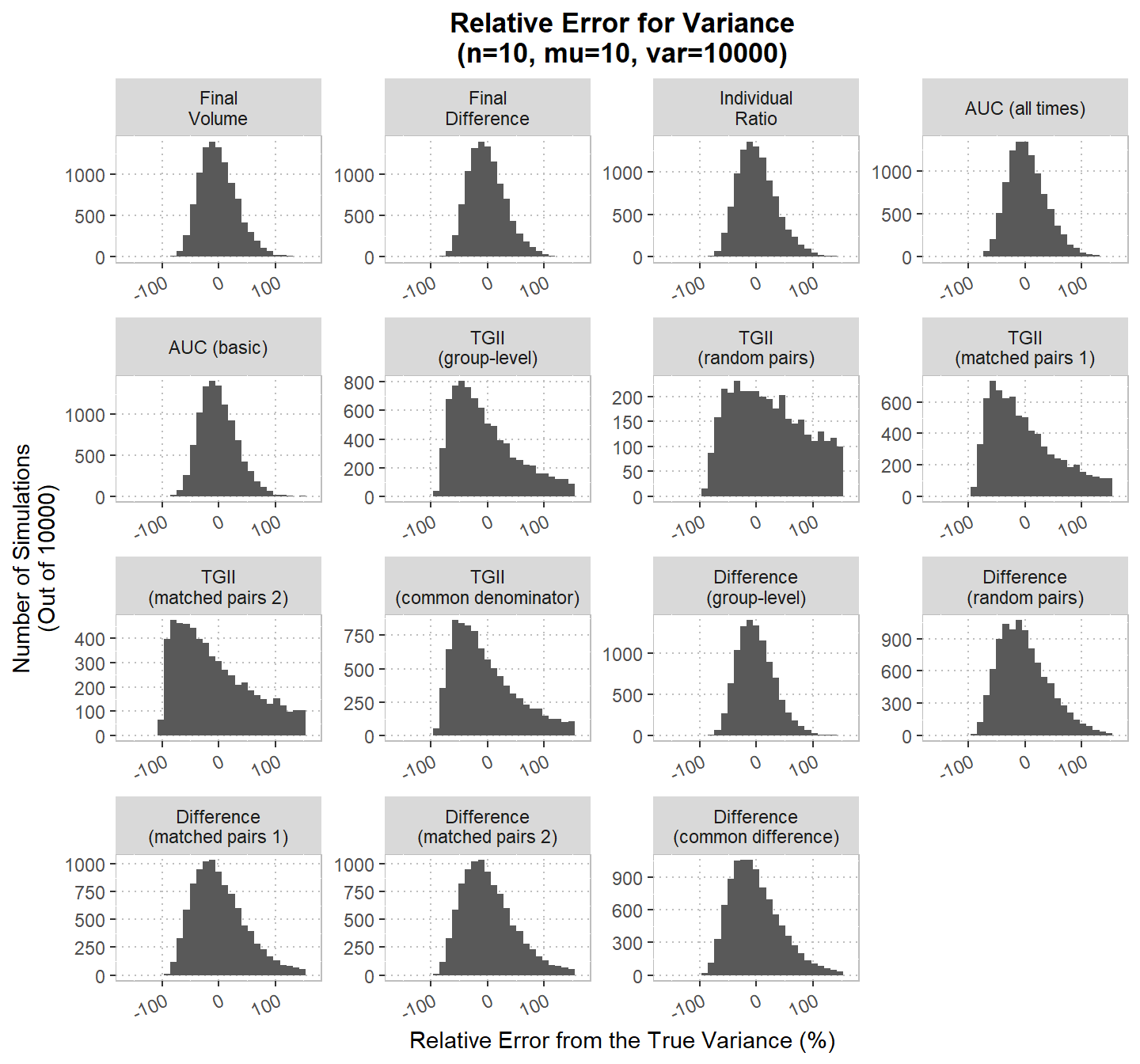


**b.**

**a.**
